# Everyday Discrimination and Mental Health Symptoms Among Hispanic and Non-Hispanic Students of Color Attending a Hispanic Serving Institution

**DOI:** 10.1089/heq.2020.0095

**Published:** 2021-05-13

**Authors:** Dylan G. Serpas

**Affiliations:** Department of Psychology, California State University, Fullerton, Fullerton, California, USA.

**Keywords:** college student health, health disparities, minority mental health, emerging adult mental health, everyday discrimination

## Abstract

**Purpose:** Discriminationas a unique psychosocial stressordisproportionately affects the mental health of communities of color as a function of systems of power and oppression. The increasing population of Hispanic undergraduates nationally warrants the importance of understanding the impact of discrimination on the mental health of students within Hispanic Serving Institutions (HSIs), which enroll the most Hispanic students across the nation. This study investigated differences in the relationship between discrimination and mental health symptoms among Hispanic and non-Hispanic students of color (SoC) to better contextualize student experiences within an HSI setting.

**Methods:** This study included 244 SoC (mean_age_=21.52, standard deviation=2.64; 65% Hispanic/Latinx; 76% female) attending a small private Liberal Arts HSI in Southern California. Participants responded to measures assessing everyday discrimination, depressive symptoms, and anxiety symptoms. Moderation analyses were performed to examine the moderating role of race/ethnicity on the relationship between everyday discrimination and mental health symptoms among Hispanic and non-Hispanic SoC.

**Results:** Both groups generally reported similar levels of everyday discrimination and mental health symptoms. Moderation analyses indicated that, after accounting for covariates, everyday discrimination was associated with more depressive and anxiety symptoms, with race/ethnicity moderating this relationship. A moderation effect was detected among respondents reporting high levels of everyday discrimination wherein Hispanic participants endorsed significantly greater depressive and anxiety symptoms.

**Conclusion:** Findings suggest that within this HSI, Hispanic students may be at greater risk of adverse mental health outcomes compared to non-Hispanic SoC when exposed to high levels of everyday discrimination.

## Introduction

Understanding factors that impact undergraduate population health is vital to promote the academic success and positive health outcomes of this population. Over 17 million students are enrolled in institutions of higher education across the United States, with about 40% identifying as students of color^a^ (SoC).^1^ The U.S. bachelor's graduation rate is 60%, and lower rates are reported for Latinx and African American students.^2^

Navigating higher education is notoriously stressful; however, expected stressors are exacerbated by experiences of discrimination. Soc are faced with additional and unexpected experiences (e.g., discrimination) compared to their nonminority counterparts, as supported by the minority stress framework.^3^ This model posits that adverse experiences targeted toward persons holding minority status(es) function as cumulative vulnerabilities and contribute to adverse psychological and physiological health consequences. These observations are a function of interpersonal-, community-, and institutional-level interactions and policies established by dominant groups.^3^

Discrimination is designated as an independent psychosocial stressor^4^ and a social determinant of health.^5^ Discrimination against SoC on college campuses is consistently reported across a variety of campus environments,^6,7^ notably among African Americans and Latinx.^8^ These adverse psychosocial experiences promote a hostile and invalidating campus environment,^9^ reduce academic performance,^10^ and are uniquely associated with greater anxiety and depressive symptoms.^11^ Although white/European Americans do report instances of racial/ethnic discrimination, its frequency and effects are not as robust compared to communities of color.^12,13^ Discrimination within higher education settings functions within a broader sociohistorical context pertaining to the structural racism and prejudice embedded within U.S. discourse. Indeed, SoC who experience high levels of race-related stress are more likely to develop mental health difficulties.^14^ This study defines discrimination as unfair or disrespectful treatment based on a prejudicial assumption^15^ and defines mental health as an absence of psychopathologies, including depression and anxiety.^16^

Mounting evidence confirms a unique relationship between experiences of discrimination and depression and anxiety.^1719^ Moreover, SoC are uniquely affected by discrimination given their minority status within an academic enclave. Several studies have confirmed associations between various types of discrimination and reduced mental health (i.e., higher anxiety and depressive symptoms) across several racial/ethnic groups of SoC.^2024^ It is important to note that while research indicates inflated rates of depression and anxiety among communities of color, these observations are attributed to the process of navigating sociohistorical bias, prejudice, and racism rooted in the United States. In sum, experiences of discrimination disproportionately affect SoC in higher education settings and function as one pathway in the development and maintenance of mental health and education disparities. These robust findings have confirmed a relationship between discrimination and mental health among SoC in higher education settings.

The nation has observed an increase in minority-serving institutions of higher education to aid in the reduction of education disparities. Federal legislation has allocated funds intended to promote acquisition, retention, and degree-attainment among SoC. For instance, Hispanic Serving Institutions (HSIs) aim to increase the number of Hispanic/Latinx^b^ college graduates. HSIs were recognized at the federal level with the enactment of the Hispanic-Serving Institutions of Higher Education Act of 1989, which provided financial assistance to institutions to enhance their ability to graduate Hispanic/Latinx students. HSIs refer to degree-granting public or private nonprofit institutions of higher education that contain minimally 25% of an enrollment ratio of undergraduate full-time Hispanic students.^25^ Notably, HSIs are defined solely by enrollment ratios and not by the institution's academic missions or pursuits.^25^ As of 2017, there were 523 HSIs that enrolled 66% of all Hispanic/Latinx undergraduates within the United States.^26^

To date, most research on campus experiences among SoC has focused on predominantly white institution (PWI) environments.^27^ SoC at PWIs face excess anxiety, stress, and isolation associated with adverse race-related campus experiences.^6,2732^ On the contrary, research among SoC experiences in HSI settings contains mixed findings. Some studies have found that Hispanic/Latinx students attending HSIs with over 50% Hispanic enrollment report a strong sense of belonging and a culturally supportive environment that facilitate positive ethnic identity development.^33,34^ While other studies report that Hispanic and non-Hispanic SoC report similar psychosocial adversities as their non-HSI attending counterparts,^35^ an observation previously reported among HSIs with below 50% Hispanic enrollment.^27^ Past research has also found levels of discrimination to be lower among Hispanic students compared to non-Hispanic SoC.^27^

The demographic makeup of an institution is implicated in the campus climate,^36^ which includes the prevalence of discrimination and its subsequent impact on mental health. Indeed, African American, Asian American, and multiracial students are more likely to enroll in HSIs compared to their white counterparts.^37^ Thus, the student body within HSIs is expected to be ethnically diverse,^37^ which may subsequently contribute to lower perceived racial marginalization and perhaps reduced minority stress. In support of this conjecture, previous research has found that attending an HSI was associated with greater academic success among Latinx, which was suggested to be attributed to HSIs providing students with a more positive and inclusive campus climate.^38^ For instance, one factor suggested to promote a positive campus climate is the presence of microclimates aimed at providing safe and inclusive spaces for SoC.^39^

Conversely, research has found high rates of prejudice against non-Hispanic groups of SoC within HSI settings, specifically African American students.^40^ Experiences of discrimination can produce a negative perception of the campus environment, which is linked to campus belongingness,^41,42^ help-seeking behaviors,^43^ and degree completion.^44^ Taken together, while findings are mixed, it is suggested that Hispanic students may report lower experiences of everyday discrimination compared to non-Hispanic SoC counterparts attending an HSI. This difference may subsequently yield better mental health outcomes among Hispanic students.

To our knowledge, no study has empirically examined the differences in the relationship between everyday discrimination and mental health among Hispanic and non-Hispanic SoC within an HSI setting. The proposed investigation intends to clarify the relationship between discrimination and mental health among Hispanic and non-Hispanic SoC enrolled in an HSI. It is hypothesized that (1) everyday discrimination will be positively associated with anxiety symptoms and depressive symptoms, and (2) race/ethnicity will moderate this relationship such that Hispanic students will report lower levels of discrimination and mental health symptoms.

## Methods

### Participants

Participants (*N*=244) consisted of 186 females (76%) and 58 males (24%) whose ages ranged from 18 to 29 years (mean=21.52, standard deviation [SD]=2.64). The sample was drawn from a private liberal arts HSI in Southern California with 46% Hispanic/Latinx enrollment. Participants were majority Hispanic/Latinx (65%), heterosexual (91%), and juniors (41%). Complete sample demographic characteristics are provided in Table 1.

**Table 1.** Sample Demographic Characteristics Among the Pooled Samples and Stratified by Ethnicity

	Pooled sample (N=244)		Hispanic (n=158)		Non-Hispanic (n=86)	
	*M* (SD)		*M* (SD)		*M* (SD)	
Age	21.52 (2.64)		21.06 (2.53)		21.31 (2.56)	
Everyday discrimination	1.10 (0.67)		1.10 (0.67)		1.08 (0.66)	
Anxiety symptoms	0.97 (0.78)		1.07 (0.83)		0.79 (0.64)	
Depressive symptoms	0.88 (0.78)		0.94 (0.82)		0.77 (0.70)	

	**Frequency**	**%**	**Frequency**	**%**	**Frequency**	**%**
Gender						
Female	186	76.2	131	82.9	55	64
Race/ethnicity						
Hispanic/Latinx	158	64.8				
African American	19	7.8			19	22.1
Asian American	14	5.7			14	16.3
Pacific Islander	6	2.5			6	7
Native American	3	1.2			3	3.5
Bi/Multiracial	37	15.2			37	43
Other	7	2.9			7	8.1
Sexual orientation						
Heterosexual	223	91.4	147	93	76	88.4
Gay	3	1.2	2	1.3	1	1.2
Lesbian	1	0.4	1	0.6	1	9.3
Bisexual	15	6.1	7	4.4	1	1.2
Other	2	0.8	1	0.6		
Class standing						
Freshman	21	8.7	9	5.7	12	14.1
Sophomore	43	17.8	28	17.8	15	17.6
Junior	98	40.5	68	43.3	30	35.3
Senior	80	33.1	52	33.1	28	32.9

M, mean; SD, standard deviation.

### Procedure

All study materials and procedures were approved by a university's Institutional Review Board before data acquisition. Participants were recruited within classroom settings in the department of psychology. Research personnel contacted instructors for prospective data collection. Instructors granted prior permission to attend their classes and recruit potential participants. Participants were verbally informed about the study and their rights as research participants. Those who agreed to participate received two consent forms to sign. Prospective participants were informed that the study sought to explore interpersonal experiences and mental health among undergraduates. Research consent forms were passed around to students who had the option to acquire and sign the document. Students signed and returned the researcher's consent form and kept the second consent form for their records. Students subsequently received the questionnaire, which was completed in less than 10min. Participants were debriefed once all surveys were compiled and returned. Consent forms and surveys were separated into two piles and placed in separate folders to maintain anonymity.

### Measures

#### Sociodemographic questionnaire

Participants provided their age, gender, race/ethnicity, sexual orientation, and class standing. The sample was composed exclusively of SoC, wherein the sample was grouped into Hispanic (*n*=158) and non-Hispanic SoC (*n*=86) discrete categories to explore primary study aims.

#### Depression Anxiety Stress Scale-21

The Depression Anxiety Stress Scale-21 (DASS-21) is a 21-item self-report measure of depressive, anxiety, and stress symptoms over the past week.^45^ The DASS-21 contains 3 subscales, including depression, anxiety, and stress, that each contain 7 items anchored on a 4-point scale ranging from 0 (did not apply to me at all) to 3 (applied to me very much or most of the time).^45^ For study purposes, only the Depression and Anxiety subscales were used. Items were averaged and no items were reverse scored. Previous studies have reported adequate psychometric properties among samples of SoC.^46,47^ Studies also support using DASS-21 subscales as independent measures of depressive, anxiety, and stress symptoms.^11^ Adequate internal consistency reliabilities were observed for the anxiety (**=0.88) and depression (**=0.92) subscales in the current study.

#### Everyday Discrimination Scale

The Everyday Discrimination Scale (EDS) was used to assess the frequency of various forms of interpersonal unfair or unjust treatment in day-to-day life.^15^ This unidimensional instrument contains 9 items (e.g., You are treated with less courtesy than other people are.) anchored on a 4-point Likert scale with response options ranging from 0 (never) to 3 (often). Responses were averaged to create an everyday discrimination index; no items were reverse scored. In the current study, the nine items produced adequate internal consistency reliability (**=0.90). The EDS has demonstrated adequate psychometric properties across racial/ethnic groups.^15^

Descriptive statistics for all measures are provided in Table 1.

### Analysis plan

All analyses were performed using SPSS version 26. Univariate and multivariate normality assumptions were screened using skewness and kurtosis, and *z* scores for depressive symptoms, anxiety symptoms, and everyday discrimination, and Mahalanobis distance were calculated using SPSS. Cases were considered univariate outliers if *z'*s>3.29, *p*<0.001 and multivariate outliers if Mahalanobis distance >16.27, *p*<0.001.^48^

Bivariate correlations were used to assess multicollinearity, and independent sample t-tests and one-way analyses of variances (ANOVAs) were performed to examine relationships between demographic characteristics and primary outcome variables to determine potential covariates.

Moderation analyses were conducted using PROCESS macro version 3.5 for SPSS version 26 (Model 1)^49^ to test for the moderating effect of race/ethnicity on the relationship between everyday discrimination and depressive and anxiety symptoms. Continuous variables were mean centered (i.e., transformed into deviation units by subtracting their sample mean to create a revised sample mean of zero). The interaction effect was probed at each level of race/ethnicity (Hispanic and non-Hispanic) using simple slopes analysis with conditioning values for everyday discrimination set at one SD below the mean (low scores), at the mean (average), and above the mean (high scores).^50^ Moderation effects are expected to account for 1% to 3% of the variability in the criterion.^51,52^ Evidence of a moderation effect is provided by a significant incremental *R*^2^. Given the difficulty with detecting moderation effects, a liberal alpha (i.e., .10; alpha_current study_ 0.10/2=0.05) is recommended.^52^

## Results

### Preliminary analyses

Bivariate correlations indicated that age was unrelated to everyday discrimination, depressive symptoms, and anxiety symptoms (*p'*s>0.05). Moreover, everyday discrimination was associated with anxiety symptoms and depressive symptoms (*p'*s<0.05), and anxiety symptoms and depressive symptoms were significantly and positively associated (*p*<0.05). Complete bivariate correlations are provided in Table 2.

**Table 2.** Bivariate Correlations Among Primary Study Variables

Variable	1	2	3	4
(1) Age		0.008	0.036	0.002
(2) Everyday discrimination			0.536^***^	0.495^***^
(3) Anxiety symptoms				0.833^***^
(4) Depressive symptoms				

^***^ *p*<0.001.

Independent sample *t*-tests indicated that between Hispanic and non-Hispanic SoC, Hispanic participants reported greater anxiety symptoms (*p*<0.05), while no differences were found in depressive symptoms or everyday discrimination (*p'*s>0.05). Moreover, in terms of gender differences, females reported more depressive symptoms and anxiety symptoms compared to males (*p'*s<0.05), but no difference in levels of everyday discrimination was found (*p*>0.05). Respondents who self-identified as lesbian, gay, or bisexual did not differ in levels of depressive symptoms, anxiety symptoms, or everyday discrimination compared to heterosexual counterparts (*p'*s>0.05). One-way ANOVAs did not reveal differences in depressive symptoms, anxiety symptoms, or everyday discrimination across levels of class standing (*p'*s>0.05). Finally, two cases exceeded criteria for multivariate outliers and were excluded from analyses (*n*=244). An absence of normality and regression assumption violations was confirmed before data analysis.

Two moderation analyses were performed to examine the moderating effect of race/ethnicity (Hispanic and non-Hispanic) on the relationship between everyday discrimination and mental health symptoms. Gender was included as a covariate for all analyses given differences between males and females in depressive and anxiety symptoms.

### Moderations

#### Depressive symptoms

The first moderation model was significant, *F*(4, 239)=24.07, *p*<0.001, explaining 29% of the variability in depressive symptoms. As hypothesized, race/ethnicity moderated the relationship between everyday discrimination and depressive symptoms, *b*_interaction_=0.32, *t*(239)=2.39, *p*=0.018, after accounting for gender differences. This interaction accounted for an additional 2% of the variability in depressive symptoms.

**FIG. 1.**
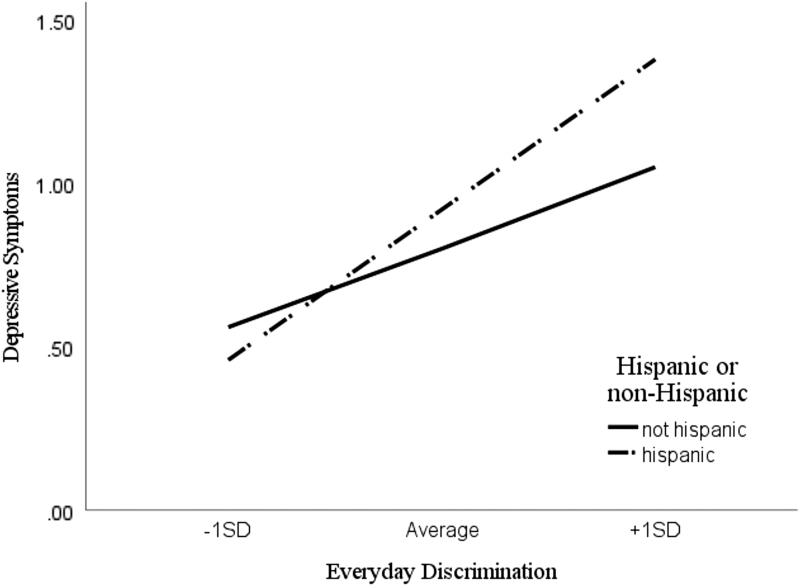
Simple slopes analysis examining the moderating role of race/ethnicity on the relationship between everyday discrimination and depressive symptoms.

Analysis of simple slopes indicated that the relationship between everyday discrimination and depressive symptoms was stronger among Hispanic students. Hispanic and non-Hispanic SoC who endorsed low everyday discrimination levels (1 SD below the mean) did not differ in depressive symptoms, *b*=0.03, *t*(239)=0.74, *p*=0.459. However, among respondents who endorsed high everyday discrimination (1 SD above the mean), Hispanic students reported significantly higher depressive symptoms, *b*=0.45, *t*(239)=2.61, *p*=0.010). It thus appears that significant differences in the relationship between everyday discrimination and depressive symptoms across race/ethnicity were only present among respondents reporting high levels of discrimination. Results are provided graphically in Figure 1.

#### Anxiety symptoms

The second moderation model was significant, *F*(4, 239)=33.97, *p*<0.001, explaining 36% of the variability in anxiety symptoms. As hypothesized, race/ethnicity moderated the relationship between everyday discrimination and anxiety symptoms, *b*_interaction_=0.37, *t*(239)=2.87, *p*=0.005, after accounting for gender differences. This interaction accounted for an additional 2% of the variability in anxiety symptoms.

**FIG. 2.**
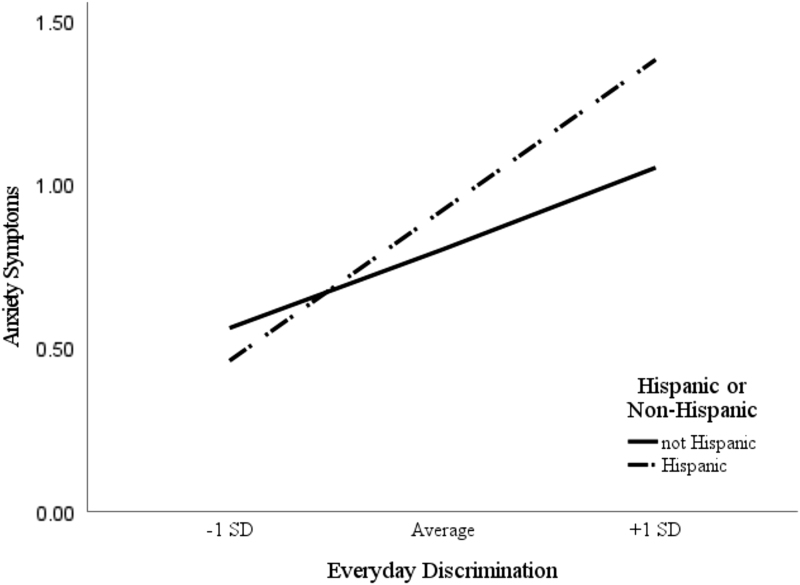
Simple slopes analysis examining the moderating role of race/ethnicity on the relationship between everyday discrimination and anxiety symptoms.

Analysis of simple slopes indicated that the relationship between everyday discrimination and anxiety symptoms was stronger among Hispanic students. Among respondents reporting low (1 SD below the mean) levels of everyday discrimination, no differences in anxiety symptoms were found between Hispanic and non-Hispanic SoC, *b*=0.03, *t*(239)=0.26, *p*=0.794. Also, among respondents reporting high levels of everyday discrimination (1 SD above the mean), Hispanic students reported significantly greater anxiety symptoms, *b*=0.46, *t*(239)=0.3.75, *p*<0.001. In conclusion, a significant moderation effect was only observed among respondents endorsing high levels of everyday discrimination. Results are provided graphically in Figure 2.

## Discussion

This study examined how the relationship between everyday discrimination and mental health symptoms differed between Hispanic and non-Hispanic SoC attending an HSI. It was first hypothesized that everyday discrimination would be associated with more mental health symptoms across the sample. This hypothesis was supported and reflects a wealth of previous studies that document similar findings among SoC.^6,14,1724^ Notably, comparable levels of discrimination and mental health symptoms were detected between Hispanic and non-Hispanic SoC. The current study sample was drawn from an HSI with 46% Hispanic/Latinx enrollment. Previous studies have reported positive academic and mental health outcomes among Hispanic/Latinx students enrolled in HSIs with greater than 50% Hispanic enrollment.^33,34^ Thus, it is possible that differences in levels of discrimination were not detected due to the institution containing below a 50% Hispanic enrollment.

There is a dearth of literature focusing on HSI and even less on how experiences within HSI settings differ between Hispanic and non-Hispanic SoC. The second hypothesis predicted that the relationship between everyday discrimination and mental health symptoms would depend on participants' race/ethnicity. While levels of discrimination between Hispanic and non-Hispanic SoC were comparable, the subsequent effect on mental health symptoms was not. Study findings revealed that among students endorsing high levels of everyday discrimination, Hispanic students reported greater depressive and anxiety symptoms compared to non-Hispanic SoC. This finding suggests that the impact of everyday discrimination for Hispanic students may confer greater risk to mental health compared to non-Hispanic SoC. This finding reflects previous studies that have suggested HSI enrollment below 50% may not provide Hispanic students with protective elements that reduce racial stress.^3335^ This finding is also contrary to other studies that have reported inflated rates of discrimination among SoC compared to Hispanic students within an HSI.^39^

This investigation supports everyday discrimination as one factor contributing to mental health and education disparities among SoC by means of promoting a hostile and invalidating campus environment,^9^ reducing academic performance,^10^ and increasing rates of anxiety depression,^11^ consistent with the minority stress framework.^3^ This study revealed that everyday discrimination was associated with significant reductions in mental health among SoC in general and that this observation was magnified among Hispanic students reporting excess levels of discrimination. Thus, it is imperative to continue examining modifiable factors that adversely impact the mental health of SoC to reduce and eliminate racial/ethnic education disparities.

### Limitations and future directions

This study contains notable limitations. Given the cross-sectional and correlational nature of the design, neither causality nor temporal stability of the relationships under study can be inferred; further studies should examine the proposed relationships across time. In addition, the discrimination measure used in this study was potentially underreported or interpreted differently across racial/ethnic groups and did not exclusively capture experiences within academic settings. Moreover, data were captured via self-report and are vulnerable to response bias. Also, subgroups among Hispanic and non-Hispanic SoC were not considered, which neglected the unique historical and contextual factors that contribute to the health of each population. Future studies should also gather larger samples to allow for additional covariates to be examined. For instance, a larger sample would have provided the opportunity to stratify Hispanic and non-Hispanic SoC by gender to detect intersectional nuances. Furthermore, cultural factors were not measured in this study; future studies should consider the role of culturally relevant factors (e.g., acculturation, immigration status) in the relationships under study. Finally, future studies should examine the proposed relationships simultaneously across multiple HSIs with varying levels of Hispanic enrollment.

## Conclusion

Findings signal the importance of assessing psychosocial adversities that disproportionately affect the mental health of SoC within higher education settings.

## Abbreviations Used

ANOVAs: analyses of variances
DASS-21: Depression Anxiety Stress Scale-21
EDS: Everyday Discrimination Scale
HIS: Hispanic Serving Institution
LGB: lesbian, gay, or bisexual
M: mean
PWI: predominantly white institution
SD: standard deviation
SoC: students of color

## Notes

^a^ The term students of color is used to refer to persons of racial or ethnic groups that experience inequitable social, economic, legal, and political power in the U.S. society because they are not European American or white (e.g., African Americans, Asian Americans, Latinx, Native Americans, Pacific Islanders, and so on).

^b^ Hispanics/Latinx may prefer to be referred to by an ancestral country of origin (e.g., Puerto Rican); however, most scientific literature omits or does not measure such information and instead used umbrella terms to refer to persons with ancestral links to countries where Spanish is the dominant language. Therefore, these terms are used interchangeably in this study.
